# Serum neuron-derived extracellular vesicle mitochondrial DNA as a potential biomarker for mild cognitive impairment due to AD

**DOI:** 10.3389/fnagi.2026.1924123

**Published:** 2026-10-15

**Authors:** Danhong Lin, Yashi Luo, Xiaxia Qiu, Peng Ye, Rongbing Chen, Wenshen Xu, Ru Zheng, Xiaomeng Fu, Kengna Fan, Dengbin Chen, Bin Yang, Longfei Chen, Qishui Ou, Xiaofeng Liu

**Affiliations:** 1 Department of Laboratory Medicine, The First Affiliated Hospital, Fujian Medical University, Fuzhou, China; 2 Department of Laboratory Medicine, National Regional Medical Center, Binhai Campus of the First Affiliated Hospital, Fujian Medical University, Fuzhou, China; 3 Department of Laboratory Medicine, Geriatric Hospital Affiliated to Wuhan University of Science and Technology, Wuhan, China; 4 Department of Blood Transfusion, Geriatric Hospital Affiliated to Wuhan University of Science and Technology, Wuhan, China; 5 Department of Neurology, The First Affiliated Hospital, Fujian Medical University, Fuzhou, China

**Keywords:** biomarkers, exosomes, mild cognitive impairment, mitochondrial DNA, neuron-derived extracellular vesicles

## Abstract

**Background:**

Mild cognitive impairment (MCI) represents the initial clinical manifestation, eventually evolving into the distinctive features of Alzheimer’s disease (AD), making its early identification crucial. Notably, mitochondrial dysfunction is an early characteristic of AD. Neuron-derived extracellular vesicles (NDEVs) can cross the blood-brain barrier, and their mitochondrial DNA (mtDNA) can reflect intracerebral lesions, showing considerable potential. In this study, we investigated the alterations in mtDNA within serum NDEVs and evaluated their potential as early diagnostic biomarkers for MCI due to AD.

**Methods:**

A total of 42 MCI patients, 49 AD patients, and 57 age-matched cognitively unimpaired (CU) controls were enrolled. Serum NDEVs were enriched and characterized, and their mtDNA levels were quantified by qPCR. ROC curve was used to analyze diagnostic performance, and the integration of multimodal biomarkers was explored.

**Results:**

Serum NDEVs mtDNA levels were decreased in patients with AD-MCI, and a significant positive correlation was observed between serum NDEVs mtDNA levels and MMSE scores in MCI patients (*r* = 0.4864, *P* = 0.0407), and a similar association was observed in the AD group (*r* = 0.3857, *P* = 0.0469). In terms of diagnostic performance, serum NDEV mtDNA demonstrated outstanding discrimination between CU controls and AD subjects, achieving an area under the curve (AUC) of 0.965 (95% CI: 0.933–0.997), with 97.92% sensitivity and 89.09% specificity. Moreover, it also demonstrated moderate efficacy in differentiating early MCI from AD (AUC = 0.736, 95% CI: 0.629–0.842; sensitivity = 72.92%, specificity = 66.67%). In exploratory analyses of clinical and metabolic factors, serum NDEV mtDNA levels were positively correlated with HCY in MCI patients, while in AD patients, NDEV mtDNA levels were elevated in those with diabetes and positively correlated with TG. Notably, integrating NDEV mtDNA with established biomarkers, including p-Tau 217, GFAP, and APOE ε4 carrier status, significantly improved the accuracy of MCI detection, highlighting its value in multimodal biomarker strategies.

**Conclusion:**

Serum NDEVs mtDNA levels were significantly reduced in patients with MCI and AD, suggesting strong potential as an early diagnostic biomarker for MCI due to AD.

## Introduction

Alzheimer’s disease (AD) is pathologically characterized by the progressive accumulation of amyloid-β (Aβ) plaques and hyperphosphorylated tau protein aggregates, with these neuropathological changes often beginning decades before clinical onset (Anijärv et al., 2025; Rafii and Aisen, 2023). However, current diagnostic approaches remain limited. When relying on combined clinical evaluation, computed tomography (CT), and cognitive assessment, diagnostic accuracy is only 61% among general practitioners and 73% even among dementia specialists (Palmqvist et al., 2024). Mild cognitive impairment (MCI) represents the initial clinical manifestation, eventually evolving into the distinctive features of AD (MCI due to AD). Consequently, MCI due to AD presents a pivotal opportunity for early detection, diagnosis, and prevention of AD (Lombardo et al., 2026). Once the disease advances into full-blown dementia, the efficacy of available treatments is markedly limited. Longitudinal cohort studies indicate that 28% of AD-MCI cases progress to AD dementia, while 45% remain clinically stable and 15% exhibit cognitive normalization (Hu et al., 2017). These findings underscore the urgent need for improved biomarkers to enable early detection and timely intervention.

In recent years, the diagnostic paradigm for AD has shifted from purely clinical assessment toward biomarker-based approaches. Technological advances have enabled a transition from cerebrospinal fluid (CSF) to minimally invasive blood-based methods, improving large-scale screening feasibility. Among blood biomarkers, extracellular vesicles (EVs), especially neuron-derived EVs (NDEVs), have attracted great interest due to their unique biological properties (Manolopoulos et al., 2025). NDEVs can cross the blood-brain barrier (BBB) and mediate intercellular communication by transporting various biomolecules (proteins, lipids, DNA, miRNA) (Kumar et al., 2024). Importantly, the neuronal specificity of NDEVs, achieved through selective isolation methods, effectively minimizes interference from non-neuronal vesicles, thereby enhancing the pathological relevance of test results (Cheng et al., 2025; Liu et al., 2025). Accumulating clinical evidence supports NDEVs-based biomarkers as a promising strategy for the early detection of neuronal dysfunction in neurodegenerative diseases.

Mitochondria are critical for cellular energy metabolism; mtDNA alterations (copy number variations, mutations) are potential biomarkers. Decreased mtDNA levels have been reported in the brain tissue and peripheral blood of AD patients versus cognitively normal individuals (Tong et al., 2024). Moreover, Mendelian randomization analyses suggest a causal contribution of mtDNA to dementia risk (Chong et al., 2022). Direct assessment of mitochondrial changes in brain tissue remains challenging; exosome-based approaches offer a minimally invasive alternative for investigating central nervous system pathology (Hirtz et al., 2023). NDEV enrichment is a promising strategy for more accurate reflection of brain pathology (Eitan et al., 2023). Emerging evidence indicates that EVs encapsulate mitochondrial components, including mtDNA and proteins (Ikeda et al., 2021; Todkar et al., 2021), and that EV-mediated transfer of these components can improve cellular bioenergetics and viability in various disease models both *in vitro* and *in vivo* (D’Souza et al., 2021; Dave et al., 2022).

In this study, we quantified mtDNA levels in serum NDEVs from individuals with AD and its prodromal stage MCI. We also measured core AD biomarkers, including serum Aβ42, Aβ40, phosphorylated tau (p-tau) 181, p-Tau 217, and glial fibrillary acidic protein (GFAP), to compare their diagnostic performance with that of NDEV mtDNA. Our aim was to evaluate the diagnostic utility of serum NDEV mtDNA for identifying MCI due to AD, with the goal of enabling earlier diagnosis and potentially slowing the progression from MCI to AD dementia.

## Materials and methods

### Study participants

A total of 275 participants were enrolled between February 2022 and October 2024 at the First Affiliated Hospital of Fujian Medical University. The flowchart showing the process participant inclusion and exclusion are illustrated in Figure 1. The diagnosis of AD and MCI were established in accordance with the criteria set forth by the 2011 National Institute on Aging and Alzheimer’s Association (NIA-AA 2011) (McKhann et al., 2011), in conjunction with brain MRI and/or PET-Aβ and/or PET-tau imaging. Serum samples were collected to test thyroid function, syphilis, folate, homocysteine, vitamin B12, fasting glucose, glycated hemoglobin, and serum lipids to exclude alternative causes of cognitive decline. According to Petersen’s MCI criteria (Petersen et al., 1999), MCI was defined by cognitive impairment reported by patients, their caregivers, or experienced clinicians, with grouping based on Mini-Mental State Examination (MMSE) scores ranging from 20 to 27. All clinical diagnoses were initial assessments, and MMSE and Montreal Cognitive Assessment (MoCA) scores were administered by experienced neurologists at the First Affiliated Hospital of Fujian Medical University. Age-matched healthy controls had no neurological or severe systemic diseases and showed normal laboratory results.

**FIGURE 1:**
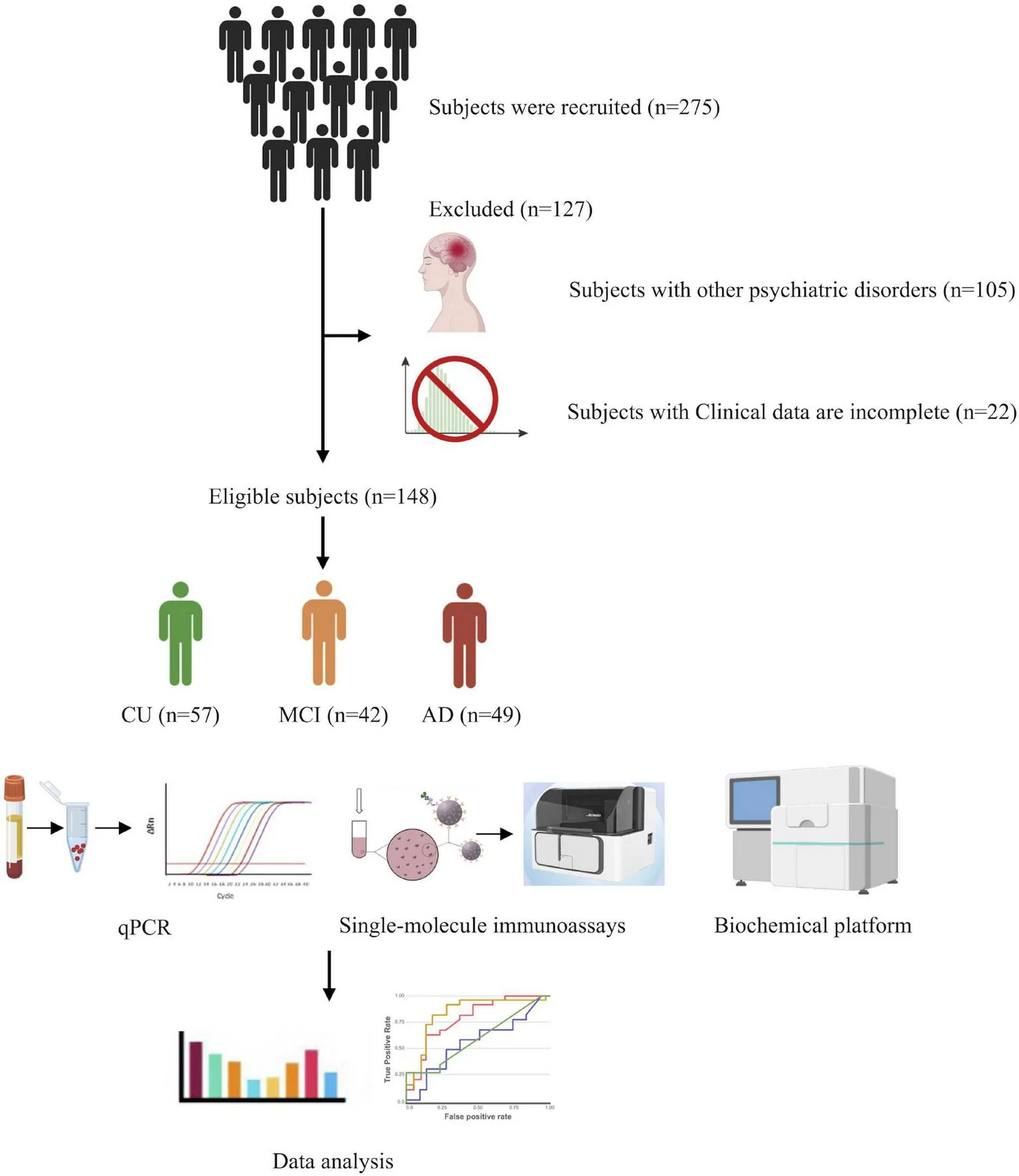
Flowchart for subject enrolment and exclusion. AD, Alzheimer’s disease; MCI, mild cognitive impairment; CU, cognitively unimpaired controls; qPCR, quantitative polymerase chain reaction.

### Serum collection and assays

Blood samples were obtained via venipuncture in the morning following a 12-h fast and collected in tubes containing coagulation activators. Serum was isolated from the blood samples by centrifugation at 2000 × *g* for 10 min at room temperature. The serum was then aliquoted into multiple polypropylene cryovials, each containing 500 μL. Serum samples were subsequently stored at −80 °C until further analysis, with care taken to avoid repeated freeze-thaw cycles. Biochemical parameters–including total cholesterol (TC), triglyceride (TG), high density lipoprotein cholesterol (HDL-C), low-density lipoprotein cholesterol (LDL-C), glucose (GLU), uric acid (UA) and homocysteine (HCY)–were measured using the cobas^®^ 8000 modular analyzer series (c702 module; Roche Diagnostics, Switzerland). Serum Aβ40, Aβ42, p-tau 181, p-tau 217, and GFAP were analyzed on the AST-Sc-Lite, a fully-auto single-molecule detection machine (Suzhou AstraBio Technol-ogy Co., Ltd., China) approved by the National Medical Products Administration (NMPA). The specific detection procedure is as follows: 25 μL of sample was mixed with capture antibody-coated magnetic beads and loaded onto the instrument. After a 6-min incubation, detection antibodies conjugated with single-molecule reporter tags were added, and the mixture was incubated at 40 °C for 4 min. The magnetic beads were then immobilized on the surface of a flow cell using magnetic capture, washed to remove unbound substances, and subjected to single-molecule imaging. Single-molecule signals were quantified, and protein concentrations were calculated based on a standard curve.

### Apolipoprotein E genotype analysis

The APOE genotypes were determined as previously described (Liu et al., 2021). Briefly, genomic DNA was extracted from peripheral blood leukocytes using a DNA extraction kit (Qiagen, Germany). DNA quality was assessed with a NanoDrop 1000 spectrophotometer (Thermo Fisher Scientific, USA). APOE genotyping was performed using a commercial DNA chip detection kit (Zhuhai Sinochips Bioscience Co., Ltd., Guangdong, China) approved by the NMPA, following the manufacturer’s instructions. Human APOE fragments encompassing two single nucleotide polymorphisms (SNP’s), rs429358 (Cys112Arg) and rs7412 (Arg158Cys), were amplified by PCR. The PCR products were then hybridized to specific probes on the chip. The hybridized products were visualized using a chromogenic agent, and the chip images were scanned to determine the genotype. The APOE genotype was further confirmed by Sanger sequencing and compared against the GenBank database (Torres-Valadez et al., 2020). The primers used for Sanger sequencing were as follows: forward primer (5′–>3′), GACCATGAAGGAGTTGAAGGCCTAC; reverse primer (5′–>3′), CTCGCGGGCCCCGGCCTGGTA. APOE ε4 carrier status was defined as the presence of at least one ε4 allele (ε4+/− or ε4+/+).

### Serum neuron-derived extracellular vesicles isolation

Total exosomes were isolated from 500 μL of serum using a commercial kit (Umbio, cat. UR52136, China), as described in our previous work (Liu et al., 2022). Serum samples were thawed in a 25 °C water bath (for frozen samples) or kept on ice immediately after collection (for fresh samples). All samples were centrifuged at 3,000 × *g* for 10 min at 4 °C, and the supernatant was transferred to a new microcentrifuge tube. The supernatant was then centrifuged at 10,000 × *g* for 20 min at 4 °C to remove cell debris and impurities. The clarified supernatant was retained for exosome isolation.

For exosome isolation, 500 μL of serum was diluted with 2 mL of pre-cooled 1× PBS and mixed with 500 μL of Blood PureExo Solution (BPS). After thorough vortexing for 1 min, the mixture was incubated at 2–8 °C for 2 h, followed by centrifugation at 10,000 × *g* for 60 min at 4 °C. The supernatant was discarded completely, and the exosome-enriched pellet was resuspended in 100 μL of pre-cooled 1× PBS. The suspension was centrifuged at 12,000 × *g* for 2 min at 4 °C, and the supernatant containing crude exosomes was collected. If a visible pellet remained, this step was repeated 2–3 times. For further purification, the crude exosome solution was loaded onto an Exosome Purification Filter (EPF) column and centrifuged at 3,000 × *g* for 10 min at 4 °C. The eluted purified exosomes were aliquoted into 50–100 μL volumes and stored at −80 °C for subsequent experiments. The total extracellular vesicle preparations were resuspended and mixed with 1 μg of L1CAM biotinylated antibody (clone 5G3; eBiosciences, cat.13-1719-82, USA) in 3% BSA for 60 min, followed by the addition of 20 μL of streptavidin agarose Ultralink resin (Thermo Fisher Scientific, cat.53116, USA) in 3% BSA and incubated at room temperature for 30 min. After centrifugation at 4 °C at 800 × *g* for 10 min and removal of the supernatant, each pellet was resuspended in cold 0.1M glycine-HCl (pH 3.0) by gentle mixing for 10 s and centrifuged at 4500 × *g* for 5 min, all at 4 °C. The supernatant was then transferred to a clean tube containing 3% BSA and 1 M Tris-HCl (pH 8.0) and gently mixed. A 50 μL aliquot of intact NDEVs was taken from each tube for Nanoparticle Tracking Analysis (NTA) and electron microscopy analysis. The resulting NDEV lysates were stored at −80 °C (Liu et al., 2022).

### Identification of serum neuron-derived extracellular vesicles

Neuron-derived extracellular vesicles were resuspended in 50–100 μL of 2% PFA, and 5 μL of NDEV suspension was applied to Formvar-carbon-coated copper grids. Two to three grids were prepared for each NDEV sample. 100 μL of PBS was dispensed onto parafilm. The copper grids (Formvar film side down) were placed on the PBS droplets for washing. The grids were then placed on 50 μL of 1% glutaraldehyde for 5 min, followed by 100 μL of ddH2O for 2 min (washed 8 times). The grids were subsequently placed on 50 μL of uranyl oxalate for 5 min and stained with methyl cellulose for 10 min on ice. The grids were positioned on the top of a stainless-steel ring on the sample stage, and excess liquid was absorbed with filter paper. The grids were air-dried for 5–10 min and then placed in a box, which was loaded into a JEM-1230 (JEOL, Tokyo, Japan) transmission electron microscope (TEM). Ultrastructural images were captured at a resolution of 200 nm. NTA measurements were conducted by injecting the samples into a NanoSight NS300 (Malvern Panalytical, UK). Particle size was evaluated using NTA 3.4 Build 3.4.4 software.

### Quantitative polymerase chain reaction detected mitochondrial DNA content

Prior to vesicle lysis, DNase I pretreatment was performed on NDEV suspensions to eliminate co-precipitated extracellular free DNA. Mitochondrial DNA was extracted in accordance with the manufacturer’s instructions and the strategy of our previous study (Liu et al., 2024) (CWBIO, cat. CW2087, China). All extracted mtDNA samples were normalized to a uniform concentration using a NanoDrop 1000 spectrophotometer (Thermo Fisher Scientific, USA) prior to qPCR analysis. qPCR was performed was performed on a 96-well full-skirted reaction plate with the QuantStudio™ 5 Real-Time PCR System (Applied Biosystems, USA) and Taq Pro Universal SYBR qPCR Master Mix (Vazyme, cat. Q712, China). Each 20 μL qPCR reaction mixture contained 10 μL Taq Pro Universal SYBR qPCR Master Mix (Vazyme, cat. Q712, China), 0.4 μL forward primer (10 μM), 0.4 μL reverse primer (10 μM), 2 μL normalized mtDNA template, and 7.2 μL nuclease-free water. Following the method described by Lazo et al. (2021) the primer sequence used is MT-ND1 (Forward primer: 5′-CCCTAAAACCCGCCACATCT-3′, Reverse primer: 5′-GAGCGATGGTGAGAGCTAAGGT-3′) and Mitochondrial gene specific primers (Forward primer: 5′-CCTTCCCCCGTAAATGATATCA-3′, Reverse primer: 5′-GCCATCTTAACAAACCCTGTTCTT-3′). The samples were subjected to an initial denaturation at 95 °C for 3min, followed by 40 cycles of 95 °C for 20 s and 60 °C for 20 s. Relative abundance for each mtDNA amplicon was quantified using a modified 2^–ΔΔCt^ approach, with normalization against the global mean Ct value (X; the average Ct across all samples for a given primer set). The calculation was performed as 2^–(X–Ct)^.

### Western blot analysis

An equal volume of exosome-specific lysis buffer (Umibio, cat. UR33101, China) was added to the NDEV sample. After thorough mixing, lysis was carried out at 4 °C for 10 min, followed by centrifugation at 12,000 rpm for 10 min at 4 °C. The supernatant was carefully collected. The NDEV protein pellets were resuspended and lysed on ice. Protein concentrations were quantified using a BCA Protein Assay Kit (Beyotime, cat. P0012S, China) according to the manufacturer’s protocols. To ensure consistent loading across all samples, all protein lysates were normalized to an equal concentration before subsequent Western blot analysis. The extracted proteins from each group were separated by sodium dodecyl sulfate-polyacrylamide gel electrophoresis and transferred to PVDF membranes. Specific antibodies were used to detect L1CAM (BD Biosciences, cat.554273, USA), Calnexin (Abcam, cat. ab22595, Britain), TSG101 (Abcam, cat. ab125011, Britain), CD63 (Abcam, cat. ab134045, Britain). Primary antibodies were incubated overnight at 4 °C. Horseradish peroxidase (HRP) – conjugated anti-mouse antibody (Beyotime, cat. A0216, China) or HRP-conjugated anti-rabbit antibody (Beyotime, cat. A0208, China) were used as secondary antibodies in blocking buffer on a shaker at room temperature for 2 h. The ECL chemiluminescence detection kit was then used for visualization. Immunoblots were quantified using the Chemidoc XRS system (Bio-Rad, USA).

### Statistical analysis

The normality of the distribution of the variables was assessed using the Shapiro–Wilk test. Normally distributed variables were compared using independent-sample *t*-test or one-way analysis of variance (ANOVA), followed by Tukey’s *post-hoc* test for multiple comparisons where appropriate. Non-normally distributed data were analyzed using the Mann–Whitney U test or Kruskal–Wallis test, with Dunn’s *post-hoc* test and Bonferroni correction applied when significant. Receiver operating characteristic (ROC) curve analysis was performed to evaluate diagnostic performance. The optimal cutoff value was determined by maximizing the Youden index, calculated as: Youden index = sensitivity + specificity − 1. Logistic regression was employed to build predictive models, and ROC curves were generated from the resulting logistic scores. Logistic regression was also performed to assess the predictive value of biomarkers after adjustment for covariates including age and sex. We performed internal validation using 1,000 bootstrap resamples and calculated the 95% confidence intervals based on the bias-corrected and accelerated (BCa) method. Spearman’s rank correlation analysis was used to evaluate monotonic associations between biomarkers, demographic indices, and clinical parameters. Outliers were identified using the ROUT method (Q = 1%) as implemented in GraphPad Prism 10, and detected outliers were excluded from statistical analyses and graphical presentations. The DeLong test was used to compare area under the curve (AUC) values between different ROC curves. Sensitivity and specificity of combined diagnostic panels were calculated based on parallel and serial testing strategies, respectively. All statistical tests were two-sided, and a *P*-value < 0.05 was considered statistically significant. All statistical analyses and graph plotting were performed using SPSS 27 (IBM Corp., Armonk, NY, USA), R (version 4.5.0, R Foundation for Statistical Computing, Vienna, Austria), and GraphPad Prism 10 (GraphPad Software, San Diego, CA, USA).

## Results

### Participant characteristics and NDEV validation and characterization

The demographic and clinical characteristics of the study cohort are summarized in Table 1. A total of 148 individuals were included, comprising 42 patients with MCI, 49 patients with AD, and 57 age-matched CU controls. In the AD group, the mean age was 68.69 ± 9.26 years, with 40.82% male and 59.18% female. The CU group had a mean age of 67.67 ± 6.59 years (45.61% male, 54.39% female), and the MCI group had a mean age of 69.50 ± 10.66 years (33.33% male, 66.67% female). No significant differences in age or sex distribution were observed among the three groups. The mean years of education were 10.73 ± 3.61 in patients with MCI and 9.63 ± 4.19 in those with AD. The APOE ε4 carrier rate was 42.11% (16/38) in the MCI group and 48.84% (21/43) in the AD group. As expected, cognitive performance differed significantly between groups. The MMSE and MoCA scores in the AD group were 7.22 ± 4.26 and 7.44 ± 6.62, respectively, which were significantly lower than the MCI group’s MMSE score (20.04 ± 4.78, *P* < 0.001) and MoCA score (13.67 ± 3.66, *P* < 0.01). Compared to the CU group, the MCI group exhibited the following significant differences (all *P* < 0.05), serum levels of TC (4.94 ± 1.26 mmol/L), TG (1.54 ± 0.70 mmol/L), LDL-C (3.32 ± 1.08 mmol/L), GLU (5.82 ± 1.47 mmol/L), and UA (332.44 ± 65.32 μmol/L) were significantly elevated. HDL-C (1.26 ± 0.24 mmol/L) levels were significantly reduced. Additionally, the AD group also showed significantly higher TG (1.57 ± 1.06 mmol/L) and GLU (5.89 ± 1.43 mmol/L) levels compared to the CU group (all *P* < 0.05).

**TABLE 1:** Clinical and demographic features of the diagnostic cohorts.

Characteristic	CU	MCI	AD
No. of individuals	57	42	49
Sex, no (%)			
Female	31 (54.39%)	28 (66.67%)	29 (59.18%)
Male	26 (45.61%)	14 (33.33%)	20 (40.82%)
Age, mean (SD), y	67.67 ± 6.59	69.50 ± 10.66	68.69 ± 9.26
Education, mean (SD), y	–	10.73 ± 3.61	9.63 ± 4.19
APOE ε4 carriers (%)	–	42.11% (16/38)	48.84% (21/43)
MMSE, mean (SD)	–	20.04 ± 4.78	7.22 ± 4.26^###^
MoCA, mean (SD)	–	13.67 ± 3.66	7.44 ± 6.62^##^
Serum			
TC, mean (SD), mmol/L	4.44 ± 0.66	4.94 ± 1.26*	4.85 ± 1.36
TG, mean (SD), mmol/L	1.12 ± 0.47	1.54 ± 0.70*	1.57 ± 1.06*
HDL, mean (SD), mmol/L	1.42 ± 0.30	1.26 ± 0.24*	1.28 ± 0.40
LDL, mean (SD), mmol/L	2.75 ± 0.62	3.32 ± 1.08*	3.12 ± 1.17
GLU, mean (SD), mmol/L	4.91 ± 0.55	5.82 ± 1.47***	5.89 ± 1.43***
UA, mean (SD), μmol/L	296.73 ± 73.28	332.44 ± 65.32*	293.61 ± 67.72
HCY, mean (SD), μmol/L	–	10.95 ± 5.64	11.78 ± 3.85
Aβ42, mean (SD), pg/mL	–	8.59 ± 5.58	10.57 ± 7.44
Aβ40, mean (SD), pg/mL	–	15.24 ± 10.89	18.71 ± 12.15
p-Tau 181, mean (SD), pg/mL	–	5.77 ± 3.37	7.62 ± 4.80^#^
p-Tau 217, mean (SD), pg/mL	–	6.17 ± 4.08	10.34 ± 7.38^##^
GFAP, mean (SD), pg/mL	–	14.48 ± 7.40	19.39 ± 9.39^#^

The normality of the distribution of the variables was assessed by the Shapiro–Wilk test. Continuous variables were compared between two independent samples using the *t*-test or the Mann–Whitney U test. Chi-square test was used to assess sex. Logistic regression models were employed to compare continuous variables between different groups before and after adjusting for covariates such as age and sex. AD, Alzheimer’s disease; MCI, mild cognitive impairment; CU, cognitively unimpaired controls; MMSE, Mini-Mental State Examination; MoCA, Montreal Cognitive Assessment; TC, total cholesterol; TG, triglyceride; HDL-C, high density lipoprotein cholesterol; LDL-C, low-density lipoprotein cholesterol; GLU, glucose; UA, uric acid; HCY, homocysteine; Aβ42, amyloid-β; p-tau217, phosphorylated tau; GFAP, glial fibrillary acidic protein.

*P < 0.05 and

***P < 0.001, compared with CU; ^#^*P* < 0.05, ^##^*P* < 0.01 and ^###^*P* < 0.001, compared with MCI.

To determine whether NDEVs were successfully enriched, NTA showed enriched particles (concentration 5.51 × 10^10^ ± 6.03 × 10^9^ particles/mL, mode diameter 132.0 ± 3.5 nm) consistent with expected EV characteristics (Supplementary Figure 1A). Western blot detected NDEV markers (L1CAM, CD63, TSG101) but not non-exosomal Calnexin (Supplementary Figure 1B). We characterized the isolated NDEVs and total EVs by Western blot, using peripheral blood mononuclear cells (PBMCs) as a control. The classical EV markers TSG101 and CD63 were detected in both total EV and NDEV fractions. In contrast, the negative marker Calnexin was not detected in either EV or NDEV samples, indicating minimal contamination from intracellular proteins. The NDEV-specific marker L1CAM was enriched in the NDEV fraction (Supplementary Figure 1C). TEM revealed vesicle-like structures with a round or cup-shaped morphology, a phospholipid bilayer, and low electron density content in the NDEV preparations (Supplementary Figure 1D).

### Serum NDEV mtDNA levels decline progressively in AD and MCI

To investigate the changes in serum NDEVs mtDNA content across different stages of AD, we quantified relative mtDNA levels using qPCR in patients with AD-MCI, patients with AD, and age-matched CU controls. As shown in Figure 2A, mtDNA levels in serum NDEVs exhibited a progressive decline with increasing dementia severity. Compared with the CU group, both the MCI and AD groups showed significantly reduced NDEV mtDNA expression (both *P* < 0.0001). Furthermore, the AD group had an even greater reduction than the MCI group (*P* = 0.0298).

**FIGURE 2:**
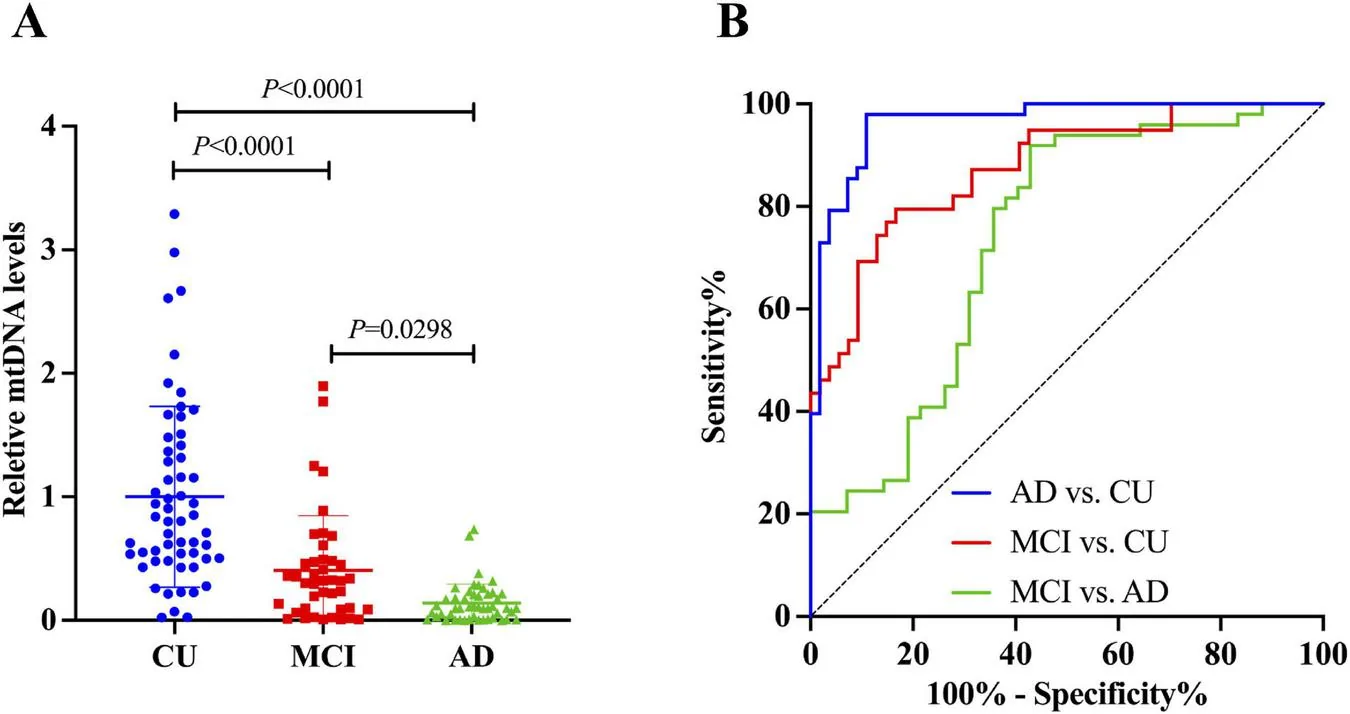
Neuron derived extracellular vesicles mtDNA levels in serum at different stages and the early diagnostic and differential diagnostic efficacy of them for AD. **(A)** The relative expression levels of serum NDEVs mtDNA at different stages (CU: *n* = 57, MCI: *n* = 42, AD: *n* = 49) were detected by qPCR. **(B)** ROC curve analysis was performed to evaluate the diagnostic efficacy of serum NDEVs mtDNA in distinguishing MCI vs. CU, AD vs. CU and MCI vs. AD (CU: *n* = 57, MCI: *n* = 42, AD: *n* = 49). AD, Alzheimer’s disease; MCI, mild cognitive impairment; CU, cognitively unimpaired controls; ROC, receiver operating characteristic.

### Diagnostic and differential diagnostic performance of serum NDEVs mtDNA in MCI and AD

To evaluate the diagnostic and differential diagnostic value of serum NDEVs mtDNA for AD, we performed ROC curve analysis and calculated the Youden index to evaluate diagnostic accuracy and sensitivity and specificity. Our results showed that serum NDEVs mtDNA exhibited good diagnostic performance for distinguishing for distinguishing CU controls MCI with an AUC of 0.875 (95% CI = 0.804–0.946), a sensitivity of 79.49%, and a specificity of 83.33% (Figure 2B). For differentiated MCI from AD, serum NDEVs mtDNA yielded an AUC of 0.736 (95% CI = 0.629–0.842), with a sensitivity of 72.92%, and a specificity of 66.67%. Additionally, it demonstrated outstanding performance in discriminating CU controls from AD dementia, achieving an AUC of 0.965 (95% CI: 0.933–0.997), a sensitivity of 97.92%, and a specificity of 89.09% (Figure 2B). All data are summarized in Supplementary Table 1.

### A comparison of serum core AD biomarkers in MCI and AD

We next compared the levels of serum core AD biomarker between patients with MCI and those with AD. There were no statistically significant differences were observed in Aβ40, Aβ42, or the Aβ42/Aβ40 ratio between the two groups (Figures 3A–C), suggesting that these Aβ-related parameters are not effective for distinguishing MCI from AD. In contrast, serum p-Tau 217, p-Tau 181, and GFAP were significantly higher in the AD group than in the MCI group (Figures 3D–F). Specifically, the AD group had mean concentrations of 10.34 ± 7.38 pg/mL for p-Tau 217, 7.62 ± 4.80 pg/mL for p-Tau 181, and 19.39 ± 9.39 pg/mL for GFAP, compared with 6.17 ± 4.08 pg/mL, 5.77 ± 3.37 pg/mL, and 14.48 ± 7.40 pg/mL, respectively, in the MCI group (all *P* < 0.05).

**FIGURE 3:**
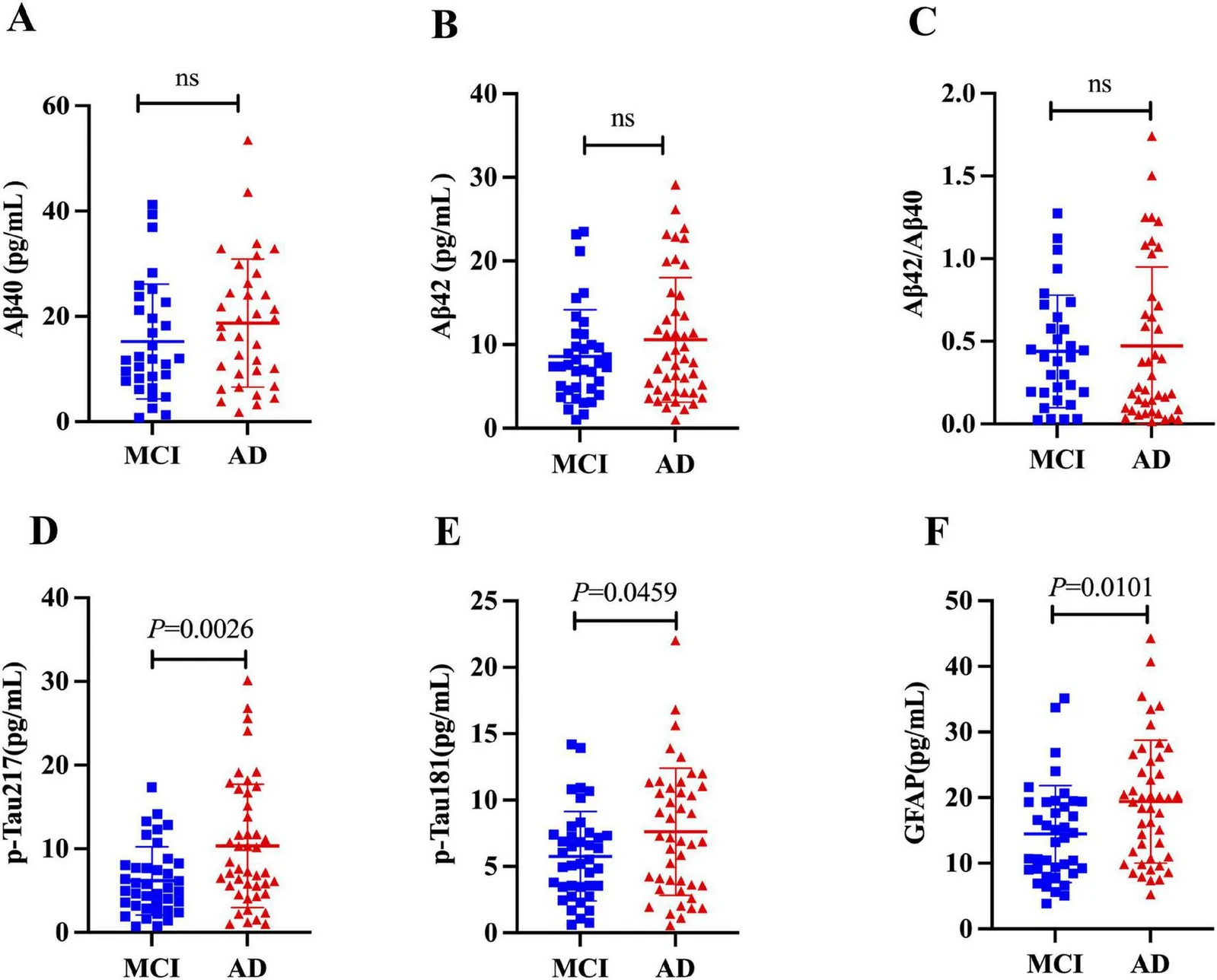
Comparison of core markers between AD and MCI. core markers **(A)** serum Aβ40 (MCI: *n* = 38, AD: *n* = 45), **(B)** serum Aβ42 (MCI: *n* = 38, AD: *n* = 45), **(C)** serum Aβ42/Aβ40 (MCI: *n* = 38, AD: *n* = 45), **(D)** serum p-Tau 217 (MCI: *n* = 40, AD: *n* = 45), **(E)** serum p-Tau 181 (MCI: *n* = 40, AD: *n* = 45) and **(F)** serum GFAP (MCI: *n* = 40, AD: *n* = 45) in MCI and AD.

### Comparison of diagnostic performance between serum NDEV mtDNA and core AD biomarkers for MCI and AD

We next compared the diagnostic performance of serum NDEV mtDNA with that of established core AD biomarkers for distinguishing MCI from AD. As shown in Figure 4A, NDEV mtDNA achieved an AUC of 0.736 (95% CI: 0.629–0.842, *P* = 0.0001), with a sensitivity of 72.92% and a specificity of 66.67%. In comparison, the core biomarkers exhibited modest performance : Aβ42 (AUC = 0.557, 95%CI: 0.432–0.682, *P* = 0.3735, Figure 4B), Aβ40 (AUC = 0.608, 95% CI: 0.473–0.742; *P* = 0.1283, Figure 4C), Aβ42/40 (AUC = 0.561, 95% CI: 0.435–0.687; *P* = 0.3442, Figure 4D), p-Tau 181 (AUC = 0.610, 95% CI: 0.489–0.732, *P* = 0.0823, Figure 4E), p-Tau 217 (AUC = 0.667, 95% CI: 0.551–0.783; *P* = 0.0092, Figure 4F), and GFAP (AUC = 0.663; 95% CI: 0.547–0.779, *P* = 0.0102, Figure 4G), these AUCs of these indicators were lower than the AUC of serum NDEV mtDNA for diagnosing AD-MCI (Figure 4H). While several biomarkers (Aβ42, Aβ42/40, p-Tau 181, GFAP) showed high specificity (>81%) and others (p-Tau 217, Aβ40) showed moderate sensitivity (>62%), none outperformed NDEV mtDNA in terms of overall diagnostic accuracy (AUC = 0.736). These findings suggest that serum NDEV mtDNA may serve as a relatively reliable early diagnostic biomarker for MCI due to AD. Detailed data are provided in Supplementary Table 2.

**FIGURE 4:**
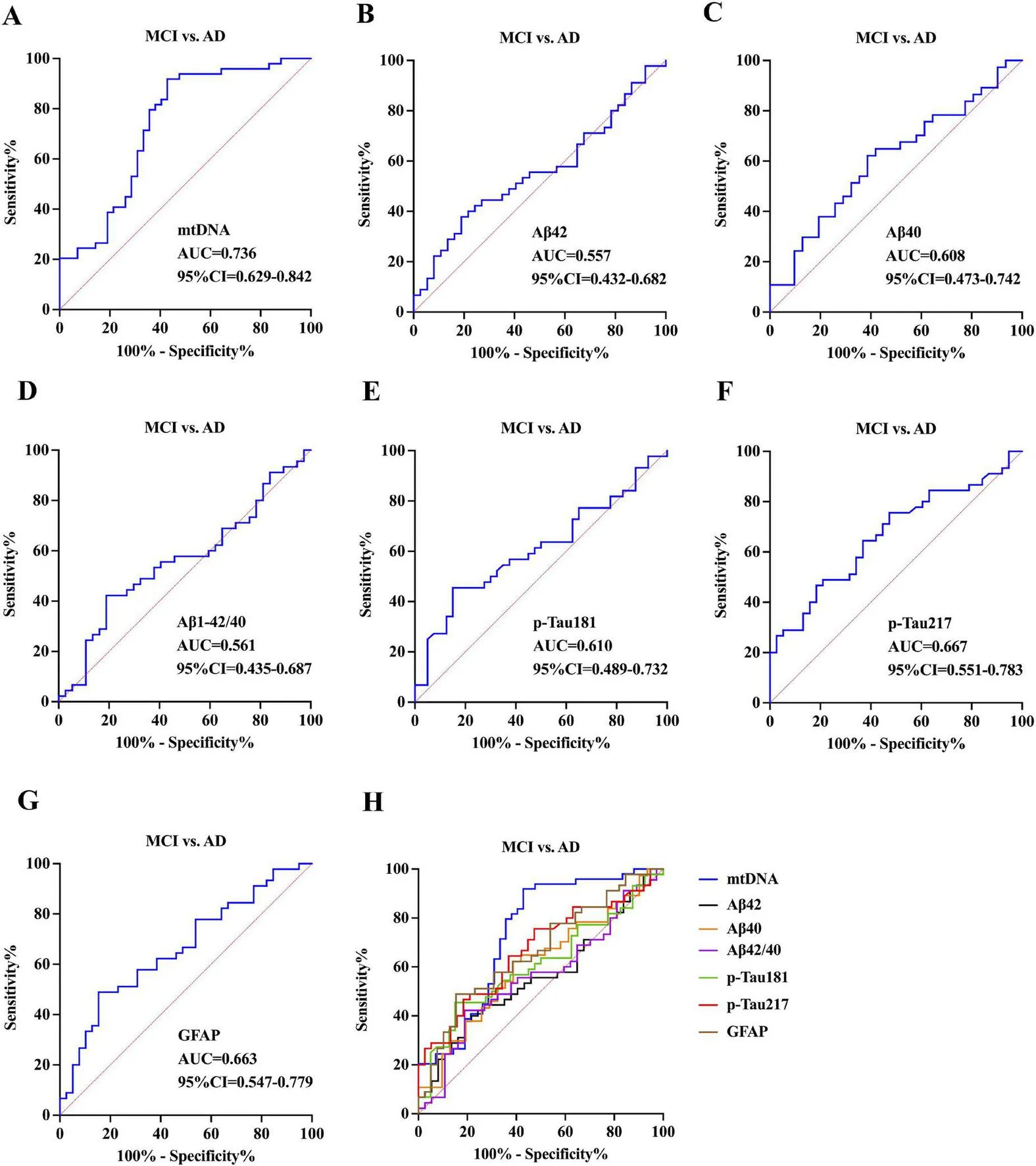
Discriminatory performance of biomarkers in distinguishing MCI from AD. Differential diagnostic efficiency of serum **(A)** NDEVs mtDNA (MCI: *n* = 42, AD: *n* = 49), **(B)** Aβ42 (MCI: *n* = 38, AD: *n* = 45), **(C)** Aβ40 (MCI: *n* = 38, AD: *n* = 45), **(D)** Aβ42/40 (MCI: *n* = 38, AD: *n* = 45), **(E)** p-Tau 181 (MCI: *n* = 40, AD: *n* = 45), **(F)** p-Tau 217 (MCI: *n* = 40, AD: *n* = 45) and **(G)** GFAP (MCI: *n* = 40, AD: *n* = 45) in MCI vs. AD. **(H)** Diagnostic efficiency of serum NDEVs mtDNA, serum Aβ42, Aβ40, Aβ42/40, p-Tau 181, p-Tau 217 and GFAP in MCI vs. AD. AUC, area under the curve; 95% CI, 95% confidence interval.

### Correlation of serum NDEV mtDNA with clinical characteristics and core AD biomarkers

We next examined whether serum NDEV mtDNA levels correlate with cognitive performance and classical AD biomarkers in patients with MCI or AD. Serum NDEV mtDNA levels were positively correlated with MMSE scores in the MCI group (*r* = 0.4864, *P* = 0.0407; Figure 5A), but no significant correlation was observed with MoCA scores (Figure 5B). No significant associations were found between serum NDEV mtDNA and any of the Aβ-related biomarkers (Aβ40, Aβ42, Aβ42/40) in either MCI or AD patients (Figure 5C) nor with p-Tau181, p-Tau217, or GFAP in MCI (Figures 5D–F). In AD group, serum NDEV mtDNA levels also showed a significant positive correlation with MMSE scores (*r* = 0.3857, *P* = 0.0469; Figure 5G). The correlation with MoCA scores was positive but did not reach statistical significance (*r* = 0.4193, *P* = 0.1356; Figure 5H). As observed in the MCI group, no significant correlations were detected between serum NDEV mtDNA and the core AD biomarkers in AD patients (Figures 5I–L).

**FIGURE 5:**
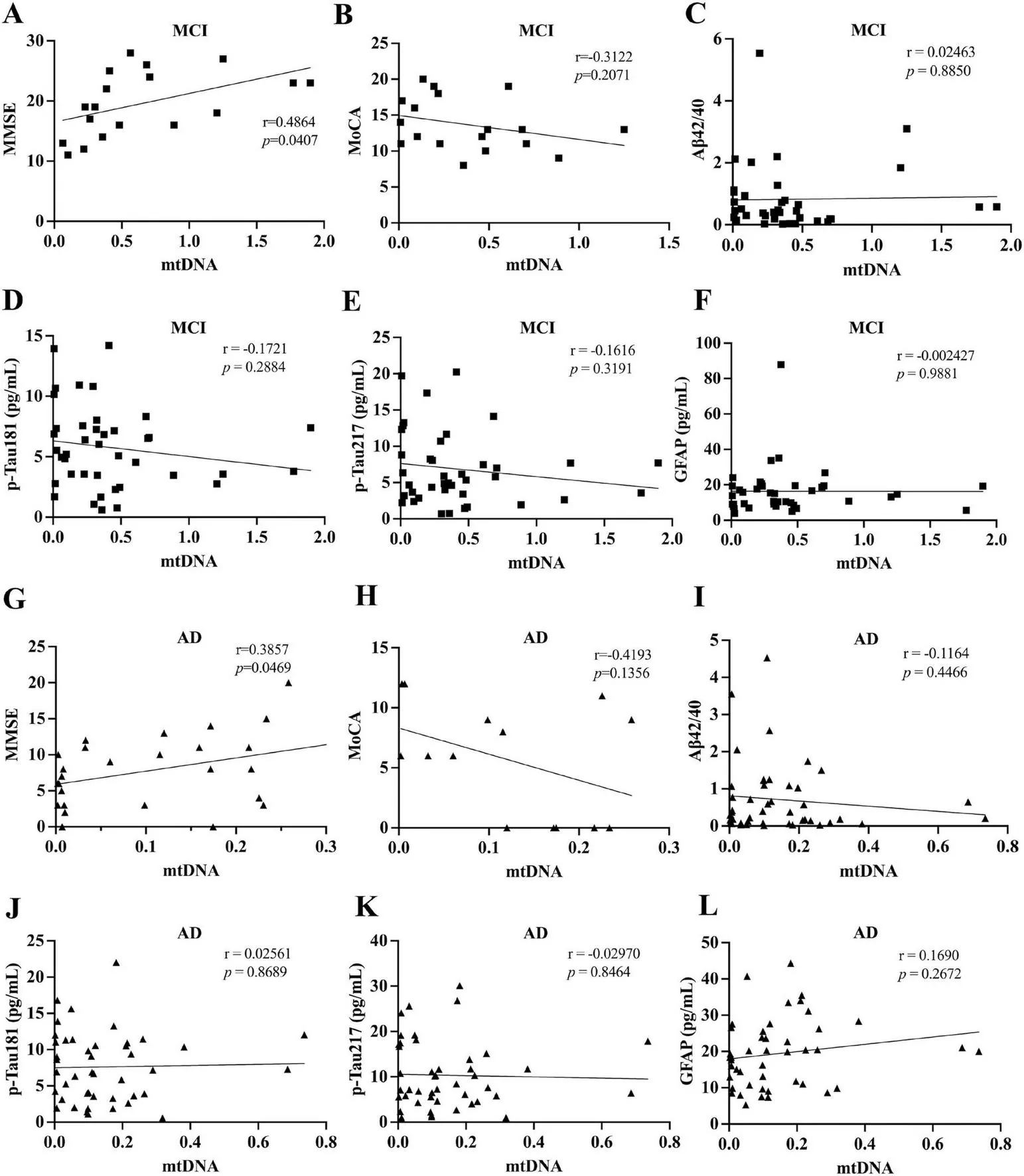
Correlation analysis of serum NDEVs mtDNA with cognitive score and AD core biomarkers in MCI and AD patients. Relationship of serum NDEVs mtDNA with **(A)** MMSE scores (*n* = 18), **(B)** MOCA scores (*n* = 18), **(C)** serum Aβ42/Aβ40 (*n* = 38), **(D)** serum p-Tau 181 (*n* = 40), **(E)** serum p-Tau 217 (*n* = 40) and **(F)** serum GFAP (*n* = 40) in MCI. Relationship of serum NDEVs mtDNA with **(G)** MMSE scores (*n* = 26), **(H)** MOCA scores (*n* = 14), **(I)** serum Aβ42/Aβ40 (*n* = 45), **(J)** serum p-Tau 181 (*n* = 45), **(K)** serum p-Tau 217 (*n* = 45) and **(L)** serum GFAP (*n* = 45) in AD.

We explored associations between serum NDEV mtDNA and lifestyle/risk factors. In MCI patients, no significant correlations were seen for gender, age, diabetes, hypertension, or TG (Supplementary Figures 2A–E), but there was a positive correlation with HCY (*r* = 0.6972, *P* = 0.0027; Supplementary Figure 2F). In AD patients, gender, age, and hypertension were not correlated (Supplementary Figures 2G,H,J), while NDEV mtDNA was higher in those with diabetes (*P* = 0.0012; Supplementary Figure 2I) and positively correlated with TG (*r* = 0.7070, *P* = 0.0002; Supplementary Figure 2K), with no HCY correlation (Supplementary Figure 2L).

### Improved diagnostic performance of multi-biomarker models incorporating serum NDEV mtDNA for AD-MCI

To identify optimal diagnostic combinations, we evaluated the performance of serum NDEV mtDNA in combination with other biomarkers for distinguishing MCI from AD. Combining serum NDEV mtDNA with Aβ40, p-Tau 217, GFAP, or APOE ε4 individually significantly improved diagnostic accuracy compared with single-indicator models. The AUC values were 0.752 (95% CI: 0.642–0.862) for mtDNA + Aβ40 (Figure 6A), 0.762 (0.659–0.865) for mtDNA + p-Tau 217 (Figure 6B), 0.783 (0.686–0.881) for mtDNA + GFAP (Figure 6C), and 0.773 (0.668–0.879) for mtDNA + APOE ε4 (Figure 6D).

**FIGURE 6:**
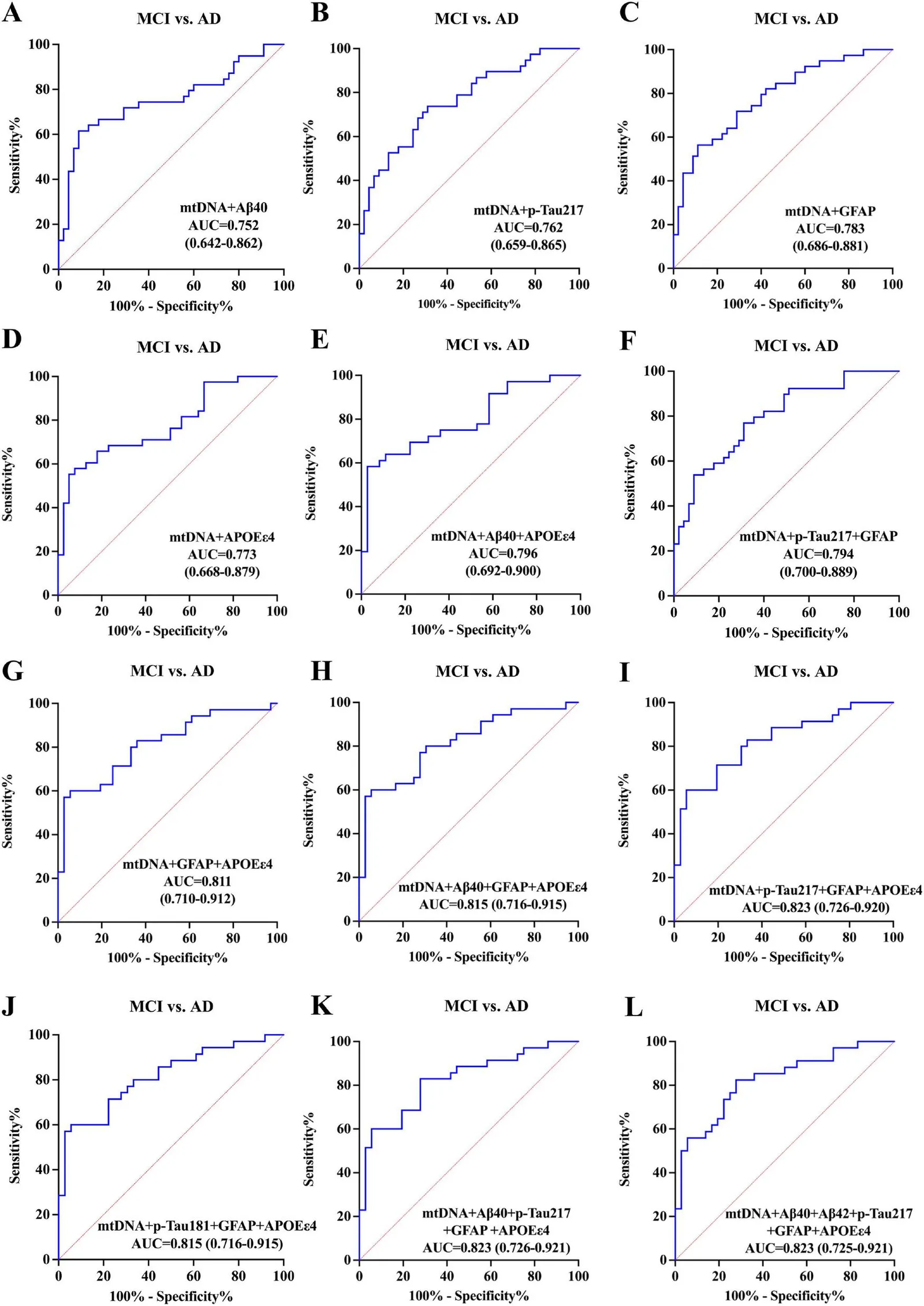
Diagnostic efficiency of serum NDEVs mtDNA combined with these biomarkers in MCI and AD (MCI: *n* = 38, AD: *n* = 43). **(A)** Diagnostic efficiency of serum NDEVs mtDNA + Aβ40 in MCI vs. AD. **(B)** Diagnostic efficiency of serum NDEVs mtDNA + p-Tau 217 in MCI vs. AD. **(C)** Diagnostic efficiency of serum NDEVs mtDNA + GFAP in MCI vs. AD. **(D)** Diagnostic efficiency of serum NDEVs mtDNA + APOE ε4 in MCI vs. AD. **(E)** Diagnostic efficiency of serum NDEVs mtDNA + Aβ40 + APOEε4 in MCI vs. AD. **(F)** Diagnostic efficiency of serum NDEVs mtDNA + p-Tau 217 + GFAP in MCI vs. AD. **(G)** Diagnostic efficiency of serum NDEVs mtDNA + GFAP + APOE ε4 in MCI vs. AD. **(H)** Diagnostic efficiency of serum NDEVs mtDNA + Aβ40 + GFAP + APOE ε4 in MCI vs. AD. **(I)** Diagnostic efficiency of serum NDEVs mtDNA + p-Tau 217 + GFAP + APOE ε4 in MCI vs. AD. **(J)** Diagnostic efficiency of serum NDEVs mtDNA + p-Tau 181 + GFAP + APOE ε4 in MCI vs. AD. **(K)** Diagnostic efficiency of serum NDEVs mtDNA + Aβ40 + p-Tau 217 + GFAP + APOE ε4 in MCI vs. AD. **(L)** Diagnostic efficiency of serum NDEVs mtDNA + Aβ40 + Aβ42+ p-Tau 217 + GFAP + APOE ε4 in MCI vs. AD.

Given the robust diagnostic performance of mtDNA when combined with Aβ40, p-Tau 217, GFAP, and APOE ε4, we then tested three-biomarker combinations (Figures 6E–G). The combination of mtDNA, GFAP, and APOE ε4 yielded the highest performance among these, with an AUC of 0.811 (95% CI: 0.710–0.912), a sensitivity of 60.0%, and a specificity of 94.4% (Figure 6G). The four-biomarker panel comprising mtDNA, Aβ40, GFAP, and APOE ε4 yielded an AUC of 0.815 (95% CI: 0.716–0.915) (Figure 6H). Replacing Aβ40 with p-Tau 217 in the panel (mtDNA, p-Tau 217, GFAP, and APOE ε4) increased the AUC to 0.823 (95% CI: 0.726–0.920), with a sensitivity of 71.4% and a specificity of 80.6% (Figure 6I). Similarly, the combination of mtDNA, p-Tau181, GFAP, and APOE ε4 achieved an AUC of 0.815 (95% CI: 0.716–0.915) (Figure 6J). Expanding to five or six biomarkers (e.g., adding Aβ40 or Aβ42) did not further improve the AUC beyond 0.823, but resulted in higher sensitivity (82.9% and 82.4%, respectively) at the cost of slightly lower specificity (72.2% for both) (Figures 6K,L). Among all panels, the mtDNA + p-Tau 217 + GFAP + APOE ε4 model showed the optimal balance between sensitivity and specificity, with diagnostic efficacy significantly superior to the single mtDNA marker (*P* < 0.001). We performed internal validation using 1,000 bootstrap resamples. After bootstrap correction, the AUC was 0.809 (95% BCa CI: 0.683–0.899), with an optimism bias of 0.014. These results indicate that the multi-marker model exhibits satisfactory internal diagnostic performance and a low risk of overfitting. Detailed data are provided in Supplementary Table 3.

## Discussion

AD encompasses a continuous pathological spectrum in which early neuronal damage precedes clinical symptoms underscoring the urgent need for novel biomarkers that enable early detection and longitudinal monitoring (Jack et al., 2024). In this study, we systematically investigate serum NDEVs mtDNA expression, diagnostic utility in MCI and AD patients, and its associations with clinical characteristics and established AD biomarkers. Findings showed a progressive decline in serum NDEV mtDNA levels paralleling cognitive impairment severity in both groups. Notably, serum NDEV mtDNA outperformed core AD biomarkers (serum Aβ40, Aβ42, Aβ42/Aβ40 ratio, p-Tau181, p-Tau217, GFAP) in distinguishing MCI from AD. Furthermore, a multi-biomarker panel integrating NDEV mtDNA, GFAP, p-Tau217, and APOE ε4 significantly enhanced the ability to predict MCI-to-AD conversion, providing a novel peripheral blood-based candidate target for AD early warning and staging diagnosis.

Literature reports have shown that a reduction in mtDNA copy number is significantly associated with a higher risk of progression from MCI to AD. In this study, we found serum NDEVs mtDNA levels were significantly lower in MCI than in CU controls, with a more pronounced decline in the AD group. Accumulating evidence suggests that mitochondrial dysfunction and aberrant mtDNA release are early events in AD. Investigations using brain tissue homogenates have generally reported reduced mtDNA levels in AD, with reductions ranging from 7% to 14% in the cerebral cortex (Klein et al., 2021) to as much as 50% in the frontal cortex (Coskun et al., 2004). In contrast, alterations in the hippocampus and cerebellum were either modest or statistically non-significant (Wei et al., 2017), indicating region-specific effects. Similarly, mtDNA levels in peripheral blood are significantly diminished in AD and exhibit a negative correlation with disease severity (Stocker et al., 2025). However, inconsistent findings across studies–attributable to small sample sizes, limited reproducibility, and ethnic heterogeneity–along with the challenges of obtaining brain tissue and the invasiveness of CSF collection, have impeded large-scale matched comparisons of central and peripheral mtDNA.

Neuron-derived extracellular vesicles present a viable solution due to their ability to traverse the blood–brain barrier and specifically indicate mitochondrial injury within the central nervous system (Andrews, 2021; Stocker et al., 2025; Zhang et al., 2023). Given that NDEVs originate from neurons, the diminished mtDNA content in serum NDEVs may directly mirror the reduced neuronal mtDNA copy number in affected brain regions.

To investigate serum NDEVs mtDNA changes in early AD, their association with core AD biomarkers (Aβ42, Aβ40, p-Tau 181, p-Tau 217), and diagnostic utility, we employed single-molecule immunoassays. Previous studies have shown that elevated blood p-Tau 181, p-Tau 217, NfL, and GFAP correlate with AD dementia risk, showing moderate 10-years predictive accuracy (Grande et al., 2025). In our cohort, serum GFAP alone distinguished AD from MCI with an AUC of 0.663. Although plasma GFAP has been reported as one of the earliest altered biomarkers in AD progression, capable of distinguishing preclinical AD, AD-MCI, and AD dementia with high accuracy (AUC > 0.85) (Shen et al., 2023), we did not observe a direct correlation between serum NDEV mtDNA and GFAP levels. Nevertheless, their combination improved discrimination between MCI and AD (AUC = 0.783). Blood p-Tau 217 has emerged as a promising AD biomarker due to its high sensitivity and specificity, even in early disease stages, outperforming other markers in distinguishing AD from CU and MCI (Li et al., 2025). However, in our study, serum p-Tau217 showed limited diagnostic utility for differentiating AD from MCI (AUC = 0.667), which is consistent with a previous report (AUC = 0.561) (Wang et al., 2024). This discrepancy may be attributable to differences in cohort size, disease stage composition, or methodological heterogeneity. Notably, serum NDEVs mtDNA demonstrated superior discrimination (AUC = 0.736) and high sensitivity compared with these core biomarkers, suggesting its potential as an early AD screening tool, particularly for high-risk populations. In terms of diagnostic performance, serum NDEV mtDNA achieved AUC values of 0.875 for distinguishing CU controls from MCI and 0.965 for CU from AD dementia, indicating good to excellent diagnostic efficacy.

For discriminating MCI from AD, the AUC was significantly higher than those of Aβ42, Aβ40, the Aβ42/Aβ40 ratio, p-Tau181, p-Tau217, and GFAP. Although p-Tau217 and GFAP have been validated as effective biomarkers for differentiating AD from prodromal MCI in previous studies (Guillén et al., 2025; Wu et al., 2025; Yang et al., 2026), the direct comparison in our cohort demonstrates that serum NDEV mtDNA exhibits superior overall discriminatory accuracy for MCI versus AD, highlighting its unique value in the staging diagnosis of AD. Notably, Aβ-related biomarkers did not show intergroup differences between MCI and AD groups in this study, which may be attributable to the susceptibility of peripheral blood Aβ to interference from peripheral metabolism and its limited sensitivity in staging during the middle-to-late phases of AD. These observations further support the complementary role of NDEV mtDNA in monitoring AD progression.

APOE4, the strongest genetic risk factor for sporadic AD, is associated with mitochondrial dysfunction in AD-vulnerable brain regions, preceding Aβ plaques, tau pathology, or cognitive decline. Neuron-specific respiratory chain alterations in APOE ε4 carriers further support mitochondrial dysfunction as an early AD hallmark (Schmukler et al., 2020). To optimize AD early detection, we constructed multi-biomarker models: mtDNA + p-Tau217 + GFAP improved diagnostic accuracy, while mtDNA + GFAP + APOE ε4 enhanced discriminatory capacity. The four-biomarker model (NDEV mtDNA, p-Tau217, GFAP, APOE ε4) achieved an AUC of 0.823, balancing the two metrics and outperforming NDEV mtDNA alone. This panel integrates multidimensional information involving mitochondrial injury, tau hyperphosphorylation, astrocytic activation and genetic susceptibility, which matches the synergistic progression of multiple AD pathological pathways. Supplementing this four-biomarker panel with Aβ40 and Aβ42 brings no incremental diagnostic improvement. This is because Aβ pathology is closely correlated with APOE ε4 status and p-Tau217 release. Besides, during MCI to AD conversion, cerebral plaque deposition makes peripheral Aβ levels plateau or even decline compensatorily, limiting their additional diagnostic value.

Moreover, our study explored the relationship between serum NDEV mtDNA levels and various clinical indicators in patients with AD and MCI. NDEV mtDNA was positively correlated with MMSE scores in both groups, linking lower levels to more severe cognitive impairment, but showed no association with Aβ or tau-related biomarkers in our cohort. These findings suggest that NDEV mtDNA may provide information that is not captured by classical Aβ/tau biomarkers, but they do not establish that NDEV mtDNA is independent of the Aβ-tau cascade. Whether alterations in NDEV mtDNA actively contribute to Aβ/tau pathology, are a downstream consequence, or represent a parallel process remains to be determined in future longitudinal and mechanistic studies. Interestingly, we identified a positive correlation between NDEV mtDNA and HCY during the MCI stage, indicating that elevated HCY levels may contribute to mitochondrial impairment in early AD. HCY is a potentially modifiable risk factor for early-stage AD, increased HCY concentrations reflect impaired metabolism of vitamin B12 and folate, which may lead to neuronal injury, cognitive decline, and an elevated risk of AD-related dementia (Zhang et al., 2025). Meanwhile, our findings indicate that during the AD stage, NDEV mtDNA levels were associated with diabetic status and TG levels. Although the precise underlying mechanism remains unclear, previous studies have demonstrated that elevated TG levels correlate with cognitive decline (Zhou et al., 2023). These observations suggest that metabolic risk factors–including hyperhomocysteinemia, diabetes, and dyslipidemia–may contribute to AD progression by modulating neuronal mitochondrial function. Our findings provide valuable clues for identifying potential metabolic intervention targets in AD.

To our knowledge, this is the first study to investigate altered mtDNA expression in serum NDEVs across the AD continuum and to demonstrate its additive diagnostic value when combined with core AD biomarkers (p-Tau217, GFAP, and APOE ε4). These findings support the translational potential of serum NDEV mtDNA for early screening and differential diagnosis of AD-MCI.

### Limitations

This study also has some limitations, including the relatively small sample size in certain subgroups limits the statistical power and generalizability of our findings. Additionally, the cross-sectional design precludes the differentiation between stable MCI and progressive MCI-to-AD conversion, and the lack of longitudinal follow-up data restricts our ability to assess the predictive value of NDEV mtDNA for disease progression. Furthermore, CSF and PET data–the gold standards for AD diagnosis–were available only for a small subset of patients, preventing direct comparison with these established biomarkers. Future studies with larger, prospective cohorts, incorporating longitudinal measurements of serum NDEV mtDNA and comprehensive hematological indicators, are warranted to validate our findings and to further elucidate the role of mitochondrial dysfunction in AD pathogenesis.

## Conclusion

In this study, we demonstrate for the first time that mtDNA levels in serum NDEVs are significantly reduced in patients with AD-MCI and in those with AD dementia, suggesting its potential as a non-invasive early-stage biomarker for MCI due to AD. Notably, the multi-biomarker model integrating serum NDEV mtDNA with p-Tau217, GFAP, and APOE ε4 significantly improved the discrimination diagnostic ability for MCI due to AD, achieving a favorable balance between sensitivity and specificity.

## Data Availability

The original contributions presented in this study are included in this article/Supplementary material, further inquiries can be directed to the corresponding authors.
