# Supplementary material for: Serum neuron-derived extracellular vesicle mitochondrial DNA as a potential biomarker for mild cognitive impairment due to AD
Source: Front Aging Neurosci. 2026 Oct 15;18:1924123. doi: 10.3389/fnagi.2026.1924123 (PMC13716654; doi:10.3389/fnagi.2026.1924123)
Supplement: Supplementary file 1 [file Data_Sheet_1.pdf]

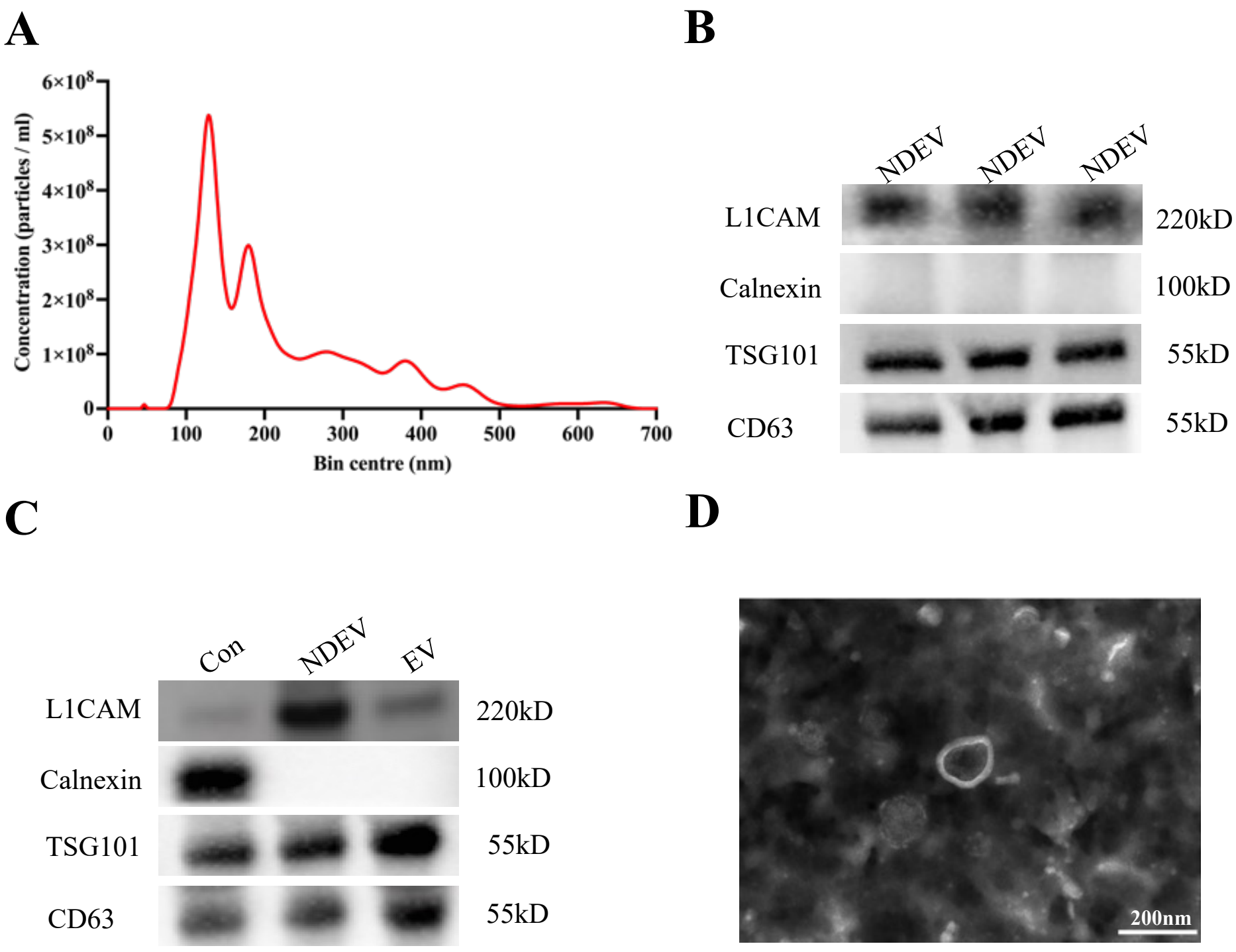

Supplemental Figure 1 Serum neuron-derived extracellular vesicles (NDEV) characterization. (A) Representative size distribution of three NDEV samples by Nanoparticle-tracking analysis (n=3). (B) Western blot analysis of L1CAM, Calnexin, TSG101 and CD63 in NDEVs (n=3). (C) Western blot detection of L1CAM, Calnexin, TSG101, and CD63 expression in Control, total EVs, and NDEVs (n=3). (D) Representative negativeative stain (Oxalate) Transmission Electron Microscopy images of NDEV samples, Scale bars: 200 nm (n=3).

Supplemental Table 1 Efficacy of serum NDEVs mtDNA for the early diagnosis and differential diagnosis of AD.

Group	AUC	95%CI	Sensitivity	Specificity (%)	*P*
AD vs. CU	0.965	0.933-0.997	97.92	89.09	<0.0001
MCI vs. CU	0.875	0.804-0.946	79.49	83.33	<0.0001
MCI vs. AD	0.736	0.629-0.842	72.92	66.67	0.0001

Diagnostic accuracy was evaluated using the receiver operating characteristic (ROC) curve analysis. The Youden index was calculated to determine the sensitivity and specificity. Abbreviations: 95% CI, 95% confidence interval; AD, Alzheimer's disease; MCI, mild cognitive impairment; CU, cognitively unimpaired controls; AUC, area under the curve.

Supplemental Table 2 The role of various markers in early diagnosis and differential diagnosis of AD.

MCI vs. AD	AUC	95%CI	Sensitivity (%)	Specificity (%)	*P*
mtDNA	0.736	0.629-0.842	72.92	66.67	0.0001
Aβ42	0.557	0.432-0.682	37.78	81.08	0.3735
Aβ40	0.608	0.473-0.742	62.16	61.29	0.1283
Aβ42/40	0.561	0.435-0.687	42.22	81.08	0.3442
p-Tau 181	0.610	0.489-0.732	45.45	85.00	0.0823
p-Tau 217	0.667	0.551-0.783	75.56	52.63	0.0092
GFAP	0.663	0.547-0.779	48.89	84.62	0.0102

Diagnostic accuracy was evaluated using the ROC curve analysis. The Youden index was calculated to determine the sensitivity and specificity.

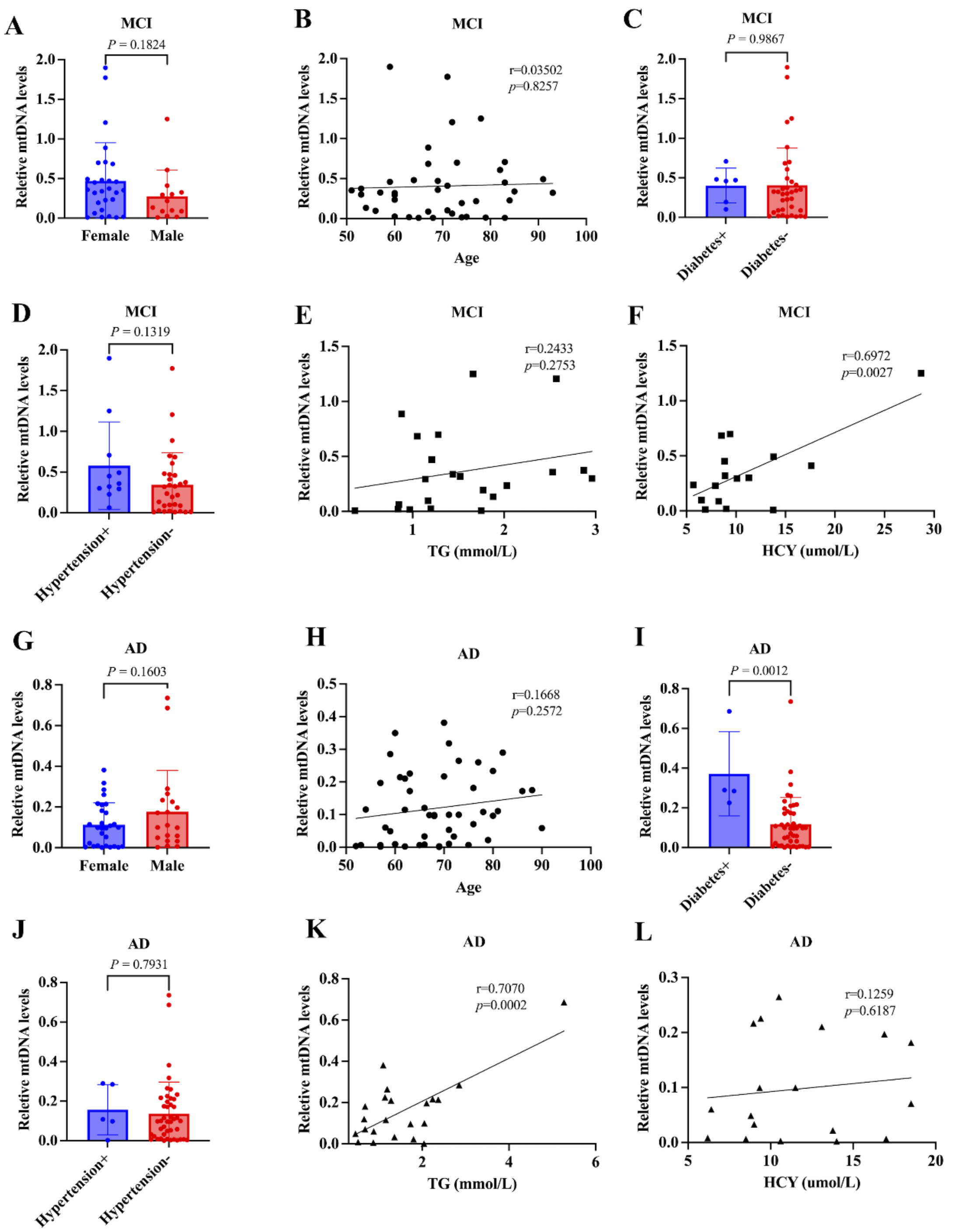

Supplemental Figure 2 Correlation analysis of serum NDEVs mtDNA with basic information and biochemical indicators in MCI and AD. (A) Comparison of serum NDEVs mtDNA expression in MCI patients with males and females (*n*=42). (B) Correlation of serum NDEVs mtDNA level with age (*n*=42). (C) Comparison of serum NDEVs mtDNA expression between MCI patients with diabetes (*n*=42). (D) Comparison of serum NDEVs mtDNA expression between MCI patients with hypertension (*n*=42). (E) Correlation of serum NDEVs mtDNA with TG level (*n*=22). (F) Correlation of serum NDEVs mtDNA with HCY level (*n*=16). (G) Serum NDEVs mtDNA expression levels in female and male in the AD

group (*n*=45). (H) Correlation of serum NDEVs mtDNA levels with age (*n*=45). (I) The mtDNA expression levels of serum NDEVs in AD patients with diabetes were compared (*n*=45). (J) The mtDNA expression levels of NDEVs in serum of AD patients with hypertension were compared (*n*=45). (K) Correlation of serum NDEVs mtDNA with TG level (*n*=22). (L) Correlation of serum NDEVs mtDNA with HCY level (*n*=19).

Supplemental Table 3 Bootstrap internal validation results of diagnostic models combining different biomarkers for discriminating MCI from AD.

MCI vs. AD	Original AUC	Bias	Corrected AUC	95% BCa CI
mtDNA + Aβ40	0.752	0.007	0.745	0.616-0.848
mtDNA + p-Tau 217	0.762	0.012	0.750	0.595-0.830
mtDNA + GFAP	0.783	0.008	0.775	0.665-0.863
mtDNA + APOE ε4	0.773	0.000	0.773	0.651-0.865
mtDNA + Aβ40 + APOEε4	0.796	0.002	0.794	0.652-0.889
mtDNA + p-Tau 217 + GFAP	0.794	0.015	0.779	0.661-0.861
mtDNA + GFAP + APOE ε4	0.811	0.013	0.798	0.661-0.890
mtDNA + Aβ40 + GFAP + APOE ε4	0.815	0.016	0.799	0.664-0.887
mtDNA + p-Tau 217 + GFAP + APOE ε4	0.823	0.014	0.809	0.683-0.899
mtDNA + p-Tau 181 + GFAP + APOE ε4	0.815	0.017	0.798	0.661-0.885
mtDNA + Aβ40 + p-Tau 217 + GFAP + APOE ε4	0.823	0.018	0.805	0.641-0.890
mtDNA + Aβ40 + Aβ42+ p-Tau 217 + GFAP + APOE ε4	0.823	0.02	0.803	0.658-0.892

Internal validation of each diagnostic model was performed using 1,000 bootstrap resamples with replacement. The corrected AUC represents the model performance after adjustment for optimism bias induced by overfitting. The 95% confidence intervals were calculated using the bias-corrected and accelerated (BCa) method.
